# Modifiable Psychological Mechanisms of Resilience Among UK Trainee and Newly Qualified Teachers

**DOI:** 10.1002/smi.70005

**Published:** 2025-01-21

**Authors:** Yun‐Han Wang, Myanna Duncan, Katherine S. Young, Colette Hirsch

**Affiliations:** ^1^ Department of Psychology Institute of Psychiatry Psychology and Neuroscience King's College London London UK; ^2^ Social Genetic and Developmental Psychiatry Centre Institute of Psychiatry Psychology and Neuroscience King's College London London UK; ^3^ South London and Maudsley NHS Foundation Trust London UK

**Keywords:** emotion regulation, newly qualified teachers, positive interpretation bias, resilience, stress, trainee teachers

## Abstract

Teaching is identified as a stressful occupation, with elevated levels of burnout among the profession. Research suggests that resilience may buffer against stress and psychological distress and potentially be a useful resource for this occupational group. This research aimed to identify mechanisms associated with trainee teachers' resilience across time. Using the cognitive model of resilience, we examined interpretation bias and emotion regulation as cognitive mechanisms associated with resilience. The study investigated whether these cognitive processes have an association with trainee teachers' resilience during teacher training and in the first year as teachers in two 1‐year longitudinal online studies. Study 1 commenced before COVID‐19 pandemic (September 2019), but ended during ongoing pandemic‐related restrictions. Study 2 was conducted as a replication study, commenced during COVID‐19 (May 2020). Resilience, short‐term stress, chronic perceived stress, and cognitive mechanisms (interpretation bias, emotion regulation) were assessed at baseline (during teacher training), with resilience and short‐term stress monitored at the 8‐month and 13‐month follow‐ups. Across both studies, cross‐sectional and longitudinal relationships were found between perceived stress, cognitive mechanisms, and resilience. Positive interpretation bias predicted trainee teachers' resilience across time, suggesting that it is likely to be a good target for interventions to promote resilience.

## 1 Introduction

Teaching is considered a stressful occupation worldwide (Thomas and Reyes 2024). Regardless of career stage, trainee and qualified teachers report high levels of stress (Agyapong et al. 2022). Common stressors reported by teachers include heavy workload and managing disruptive pupil behaviour (Tait 2008). Given that these stressors will be ongoing, unsurprisingly teachers tend to report high rates of burnout, which is a psychological syndrome resulting from prolonged exposure to chronic occupational stressors (Maslach, Jackson, and Leiter 1996). According to Chang (2009) and Durr, Chang, and Carson (2014) burnout in teachers relate to organisational, individual, and transactional factors. Organisational factors are contextual stressors such as lack of student motivation, time pressure, and low pay. Individual factors involve personality and demographic characteristics such as age, gender, and years of teaching. Transactional factors, such as the beliefs and judgements teachers make when teachers encounter stressors, are a link between organization and individual factors.

In the United Kingdom (UK), teachers report greater levels of stress and lower work‐life balance compared with other occupations (Busby 2019). Currently, there is a shortfall in recruitment and retention of teachers. Indeed, teacher training recruitment was 38% under target in 2023 (Maisuria et al. 2023), and around 30% of teachers leave the profession within the first five years (Department for Education 2023). Poor teacher retention not only contributes to a shortage of staff, but could also be related to poor student achievements (Gibbons, Scrutinio, and Telhaj 2021). While teachers' intention or decision to leave the profession may be attributable to work stress or personal issues, resilient teachers are more likely to thrive, teach effectively, manage stressors across different domains of life, and are less likely to leave the profession (Arnup and Bowles; 2016). Given this, fostering resilience in members of the teaching profession should take place early, such as among trainees or new teachers (e.g., Mansfield et al. 2016).

There are many conceptualisations of resilience. Harms et al. (2018) view resilience as a personality that adapts well after negative events, whereas Gu and Day (2007) view it as more dynamic, which manifests differently across situations and time. Rutter (1990) suggests that resilience can be developed through successful interactions between various processes and mechanisms, which opens the door to identifying processes that can be targeted to enhance resilience. Fletcher and Sarkar (2013) reviewed and critiqued current resilience definitions and found that most resilience definitions are unidimensional, such that they solely conceptualized resilience as a trait. The authors addressed the limitations of previous definitions and developed a more comprehensive definition. They define resilience as ‘*the role of mental processes and behaviour in promoting personal assets and protecting an individual from the potential negative effect of stressors*’ (p. 16). This definition is considered to be more holistic, as it encompasses two conceptualisations of resilience, the trait and process approach (Robertson et al. 2015) and considers the context and temporal dependent nature of resilience. Rather than solely capturing resilience as a stable personality‐like characteristic of coping with stressful encounters effectively, which is often assessed through the availability of coping resources to adapt to stressors and the ability to handle emotions/stress responses, the addition of capturing resilience as a process detects potential changes in resilience over time. Hence, in keeping with Fletcher and Sarkar's (2013) refined definition, we conceptualize resilience multidimensionally, having aspects of both a trait and a process.

To understand what factors may promote resilience among trainee teachers, we searched for literature on mechanisms and processes associated with resilience. A cognitive model of resilience (Parsons, Kruijt, and Fox 2016) suggests that the impact of resilience on stress levels operates via cognitive processes, in particular that cognitions/affective responses, cognitive biases, and executive function may underlie the resilience process. Based on the association between selective information processing biases and emotional vulnerability (Mathews and MacLeod 2005), adaptive cognitive biases have been proposed as mechanisms to foster resilience (Parsons, Kruijt, and Fox 2016). Kaleta et al. (2023) demonstrated that a more positive interpretation bias (the tendency to draw more positive conclusions from ambiguous situations) was associated with higher levels of resilience cross‐sectionally in general practitioners, who are in a highly stressful occupation. Longitudinally, Gordon et al. (2022) found that a positive interpretation bias predicts increased resilience 6 months later in breast cancer survivors, a population living with stress around recurrence.

Apart from positive interpretation bias, another mechanism associated with resilience is emotion regulation. Emotion regulation is defined as one's emotional response tendency or strategy after the evaluation of emotional cues (Gross and John 2003). In Parsons et al.'s (2016) cognitive model of resilience, information processing biases such as interpretations could influence subsequent emotion regulation strategies and responses, which could be adaptive or maladaptive. According to Gross (2002), there are two main strategies of emotion regulation; one is cognitive reappraisal (henceforth termed reappraisal), which involves re‐evaluating an emotion‐eliciting situation to change the emotional impact. The other is expressive suppression (henceforth termed suppression), which is the inhibition of emotion‐expressive behaviour or feelings to modulate the emotional response. Given that these two strategies both have the overarching aim to down‐regulate negative emotions, and that they are the most commonly used techniques (Gross 2002), these two emotion regulation strategies were chosen to be assessed in this study.

Johnson, Willis, and Evans (2019) suggested that resilience operates through an evaluative mechanism that allows resilient individuals to perceive potential stressors as less threatening. Xie et al. (2021) found that reappraisal is positively associated with resilience in teachers. By contrast, suppression has often been viewed as a maladaptive emotion regulation strategy (Gross and John 2003). Past research found that suppression is positively associated with emotional exhaustion and negatively associated with teaching satisfaction (Yin, Huang, and Wang 2016). In general, suppression has been suggested to be a more cognitively demanding (Gross and John 2003) process than reappraisal, as individuals who suppress are more likely to ruminate, leading to more sustained negative mood states. At present, it is unclear whether emotion regulation predicts trainee teachers' later resilience.

Stress has been suggested as a factor that elicits resilience, and moderate levels of stress can promote one's resilience to deal with subsequent stressors (e.g., Liu 2015). This mechanism, known as stress inoculation (Meichenbaum 2007), can promote resilience among those who work in high‐stress occupations, such as healthcare workers, police, and firefighters. However, exposure to long‐term stressful situations could be associated with greater psychological distress and lower resilience (Alatawi, Morsy, and Sharif 2022). It therefore remains unclear how trainee teachers' perceived levels of stress along with cognitive processes contribute to their resilience.

Norris, Tracy, and Galea (2009) emphasises that in order to understand resilience as a type of trajectory, long‐term monitoring of how one responds to stress over time is needed. Mansfield et al. (2016) proposed that some resilience related factors might be more critical in certain contexts or stages of teaching. For example, supportive networks could be more important during early stages of teaching. Given that stress associated with teaching is enduring, it is important to identify factors that promote teachers' resilience across different contexts and over time. Therefore, to understand what factors promote resilience among trainee teachers across teacher training and the initial teaching career, this study included two year‐long longitudinal studies. To date, most resilience research (e.g., de la Fuente et al. 2021; McKinley et al. 2020) adopted a cross‐sectional design, which hinders the investigation of the dynamic and context‐dependent nature of resilience under stressful contexts (Crane et al. 2012). We think that using a longitudinal approach will facilitate the understanding of what modifiable psychological mechanisms/processes found in teachers can predict and maintain teachers' future resilience across time and under different contexts.

Two studies were conducted to investigate whether interpretation bias, emotion regulation, and perceived stress are mechanisms associated with trainee teachers' resilience, and whether they predict future resilience. Study 1 assessed resilience and the mechanisms at the start of teacher training, and examined subsequent resilience 8 months later, near the end of training (after COVID‐19 started), as well as 13 months later at the start of the first teaching year. Study 2^1^ assessed the same processes initially during the final months of training, and then assessed resilience during the first year of teaching, specifically 8 and 13 months later replicating the time between follow up assessments in Study 1. However, due to the timing of the COVID‐19 outbreak in the middle of the school year, the context of assessment time points in Study 2 was not the same as Study 1, with assessment 1 conducted during the COVID‐19 pandemic.

### 1.1 Study Aims

1. Explore the correlational relationships between baseline perceived stress, positive interpretation bias, emotion regulation, and resilience in trainee teachers.
2. Explore the shared and unique contributions of variance in baseline (T1) perceived stress, positive interpretation bias, and emotion regulation on trainee teachers' resilience 8 months later (T2).
3. Explore the shared and unique contributions of variance in baseline (T1) perceived stress, positive interpretation bias, and emotion regulation on trainee teachers' resilience 13 months later (T3).

### 1.2 Hypotheses

Both studies had the same hypotheses, as follows:

H 1 *Positive interpretation bias would be positively correlated with resilience and negatively correlated with perceived stress. Adaptive emotion regulation (more reappraisal and less suppression) would be significantly correlated with lower levels of perceived stress, as well as greater levels of resilience among trainee teachers.*

H 2 *Lower baseline perceived stress, greater positive interpretation bias, and adaptive emotion regulation, as well as the combination of these variables would significantly predict trainee teachers' resilience 8 months later.*

H 3 *Lower baseline perceived stress, greater positive interpretation bias, and adaptive emotion regulation, and the combination of these variables would significantly predict trainee teachers' resilience 13 months later.*

## 2 Method

### 2.1 Design

#### 2.1.1 Cross‐Sectional and Longitudinal Study Design

We first followed the trait approach to examine what individual modifiable mechanisms of resilience were associated with qualified and trainee teachers cross‐sectionally. Then, we assessed the resilience process by examining the predictive power of a range of mechanisms in trainee teachers' future resilience via prospective and longitudinal monitoring.

#### 2.1.2 Teacher Training System in the UK

In the UK, a qualified teacher status (QTS) is required to teach in most primary and secondary schools. Individuals could obtain QTS via undergraduate or graduate teacher training routes (e.g., School Direct; Post Graduate Certificate in Education, PGCE). The present study focused on trainee teachers enrolled in full‐time graduate teacher training routes, which lasts 1 year. Within this training period, trainee teachers enrolled in PGCE programs typically complete two teaching placements, while trainee teachers enrolled in school‐based programs will immerse themselves in the school setting from the very beginning. Upon obtaining QTS, trainee teachers will work as Newly Qualified Teachers (NQTs) for a year, known as a NQT year.

#### 2.1.3 Data Collection Timeframes

In Study 1, we have designed the baseline assessment (T1) to take place at the start of training, the 8‐month follow‐up (T2) was delivered during final teaching placement,^2^ and the 13‐month follow‐up (T3) was delivered at the start of the Newly Qualified Teacher year^3^ (NQT). Study 2 followed a similar design with a later start point, the baseline assessment (T1) was 8 months after teacher training commenced which fell within the final placement period,^4^ the 8‐month follow‐up (T2) was during the second NQT term,^5^ and the 13‐month follow‐up (T3) took place at the end of the NQT year (Supplementary Table S1).

### 2.2 Participants

#### 2.2.1 Study 1

A total of 108 trainee teachers were recruited via teacher training course providers, education charities, and social media. Participants were UK‐based trainee teachers enrolled in a 1‐year full‐time training program that led to Qualified Teacher Status (QTS).

Participants who did not complete the full baseline survey (*n* = 34) or had poor performance (*n* = 2) on the Recognition Test^6^ (RT; Hirsch et al. 2021) were excluded from analyses. The number of participants retained in the subsequent follow‐up timepoints reduced (Time 2 *n* = 55, Time 3 *n* = 48).

#### 2.2.2 Study 2

Using the same recruitment method and criteria as Study 1, 179 trainee teachers were recruited (Table 1). Individuals who did not complete the full baseline survey (*n* = 53) and those with ineligible data from the RT (*n* = 2 poor task performance; *n* = 1 misunderstood instructions) were excluded from analyses. The number of participants retained in the subsequent follow‐up timepoints also reduced (Time 2 *n* = 85, Time 3 *n* = 68).

**TABLE 1:** Participant characteristics in study 1 and 2 at time 1.

Demographic variable	Study 1 (*N* = 74)	Study 2 (*N* = 123)
Age (SD)	*M* = 27.13 (7.07)	*M* = 28.50 (8.30)
Gender		
Male	28%	18%
Female	72%	82%
Training route		
University‐based	72%	62%
School‐based	28%	38%
Type of training		
Primary teacher	36%	38%
Secondary teacher	64%	62%

### 2.3 Measures

#### 2.3.1 Resilience

Resilience was measured using the 25‐item Connor‐Davidson Resilience Scale (CDRS; Connor and Davidson 2003). The scale can be used to assess one's general degree of resilience, as well as changes in resilience (e.g., stress‐management or resilience‐building programmes, psychotherapy for PTSD), enabling the examination of resilience as a trait and a process. The CDRS measures five factors: (1) personal competence, (2) trust in one's instincts, (3) positive acceptance of change, (4) control, and (5) spiritual influences. Participants rated statements (e.g., “I am able to adapt when changes occur”) on a 5‐point Likert scale ranging from 0 (*not true at all*) to 4 (*true nearly all the time*). For the scope of this study, a total resilience score was computed, with higher scores (scores could range from 0 to 100) indicating greater resilience. The internal consistency was good (*α* = 0.86 to 0.90).

#### 2.3.2 Interpretation Bias

The Recognition Test (RT; Mathews and Mackintosh 2000; Hirsch et al. 2021) and the Scrambled Sentences Test SST (Wenzlaff and Bates 1998; Hirsch et al. 2021) are two commonly used interpretation bias measures that are suitable for studies conducted via the internet, as they both does not require a speeded response. Previous research (e.g., Krahé et al. 2019; Hirsch et al. 2021) has included both tasks within a study when examining interpretation bias. The RT captures the more explicit and conscious components of interpretation bias as it allows a longer time for reflection without time constraint (Sfärlea et al. 2020). In contrast, the time constraint and cognitive load component of the SST has been suggested to capture more automatic components of interpretation bias (Wenzlaff and Bates 1998). Each measure has its strengths and limitations (e.g., the RT more subject to recall bias, the SST more subject to demand characteristics; see Schoth and Liossi 2017 for a review). It remains unclear whether the association between interpretation bias and resilience is evident on both types of measures. Therefore, the RT and SST will both be used to assess interpretation bias in the present study.

For the RT, participants were asked to read ambiguous worry scenarios and answer comprehension questions. An example scenario would be as follows: ‘Your Workload — Lately, you have been finding it difficult to get all your work done on time. As you mull over the possible reasons for this, you think you know why you are finding it hard’. Following the presentation of the scenario, participants would be asked a comprehension question to ensure they read the scenario. Later, participants rated statements on the degree to which the statement was similar in meaning to the original scenario. The statements included two target statements (e.g., “You think you will have enough time to complete all of your set paperwork”); one was a positive interpretation and the other negative. Positive interpretation bias was indexed by an interpretation bias index, where mean ratings of negative targets were subtracted from mean ratings of positive targets. Higher scores (scores could range from −1 to 1) indicate a more positive interpretation bias.

For the SST, participants formed grammatically correct sentences using five of the six words presented in random order, while remembering a string of six digits presented earlier. The words could be unscrambled to form a sentence of positive (i.e., I have succeeded at life) or negative valence (i.e., I have failed at life). Participants were asked to unscramble 20 sentences in five minutes. The interpretation bias index score was calculated by dividing the mean number of sentences with a positive valence by the mean number of grammatically correct sentences. Greater scores (scores could range from −1 to 1) indicate greater positive interpretation bias.

#### 2.3.3 Emotion Regulation

The Emotion Regulation Questionnaire (ERQ; Gross and John 2003) is a 10‐item self‐report measure of cognitive reappraisal (6 items; e.g., “I control my emotions by changing the way I think about the situation I'm in”) and suppression (4 items; e.g., “I control my emotions by not expressing them”). Participants answered questions on a 1 (*strongly disagree*) to 7 (*strongly agree*) scale. The scores for the cognitive reappraisal subscale could range from 6 to 42. The scores for the suppression subscale could range from 4 to 28. For both subscales, a higher score indicates greater utilisation of that emotion regulation strategy. The internal consistency of the reappraisal (*α* = 0.80–0.87) and suppression subscales (*α* = 0.82–0.84) were good.

#### 2.3.4 Stress

Two scales were employed to assess stress. Given that resilience could be dependent on short‐term stressors, participants' short‐term stress response was monitored by the seven‐item stress subscale from the Depression Anxiety Stress Scales—21 (DASS‐S; Lovibond and Lovibond 1995). Participants indicated how much each statement (e.g., “I found it hard to wind down”) applied to them over the past week on a 0 (*did not apply to me at all*) to 3 (*applied to me very much*, *or most of the time*) scale. The scores would range from 0 to 21, with higher scores indicating greater levels of short‐term stress response. The internal consistency of the stress subscale was good (*α* = 0.86 for both studies). The 10‐item Perceived Stress Scale (PSS; Cohen, Kamarck, and Mermelstein 1983) was used to assess long‐term perceived stress. The scores would range from 0 to 40, with greater scores indicating greater levels of long‐term perceived stress. Participants rated their thoughts and feelings (e.g., how often have you been upset because of something that happened unexpectedly?) over the past month on a 0 (*never*) to 4 (*very often*) scale. The internal consistency was good (*α* = 0.86 to 0.91).

### 2.4 Procedure

The study was approved by the university's research ethics committee. Participants completed demographic questions (i.e., gender, age, training route, type of training), the CDRS, PSS, DASS‐S, ERQ, SST, and RT at baseline. Follow‐up surveys consisted of the CDRS, PSS, and the DASS‐S. Participants were entered into a prize draw for a chance to win gift vouchers (1 of 2 £100, or 1 of 6 £50) for the baseline survey, and were paid £6 and £10 in vouchers, respectively, upon completion of the follow‐up surveys.

### 2.5 Sample Size

Using the minimum sample size of 10 participants per predictor variable (Field, Miles, and Field 2012), the sample size required was 60 participants (covariates: age, gender; predictors: perceived stress, positive interpretation bias, reappraisal, suppression). Both studies fulfiled the requirement and further participants were recruited to minimize issues with attrition.

### 2.6 Data Analysis

Data analyses were performed using R version 4.1.0. Independent *t* tests examined differences in participants' baseline variables across both studies. The same set of analyses were carried out separately for each study. A one‐way repeated‐measures analysis of variance (ANOVA) assessed differences in stress response, perceived stress, and resilience across Time (T1, T2 & T3), with Bonferroni correction [*p* value = alpha (0.05)/number of tests (3) = 0.016]. Bivariate correlations examined the relationship between baseline variables, with Bonferroni correction applied [*p* value = alpha (0.05)/number of variables (7) = 0.007]. Hierarchical regression analyses examined the shared and unique contribution of variance in baseline covariates (i.e., age, gender) and predictor variables (i.e., perceived stress, interpretation bias, and emotion regulation) on trainee teachers' resilience at T2 and T3. Two separate models were conducted, each using a different interpretation bias measure. Research has found inconsistent relationships between age, gender, and resilience (Campbell‐Sills, Cohan, and Stein 2006); thus, they were entered as covariates in step 1. The covariates and predictors were entered in step 2.

## 3 Results

### 3.1 Means and Standard Deviations

Tables 2 and 3 outline the means and standard deviations of baseline variables across both studies (See supplementary Tables S2–S5 for summary statistics in Time 2 and Time 3^7^). Participants in Study 2 reported significantly higher levels of resilience [*t* (195) = 1.98, *p* = 0.05, *Hedges' g* = 0.29] and higher positive interpretation bias scores from the RT [*t* (193) = 2.18, *p* = 0.03, *Hedges' g* = 0.32] compared to participants in Study 1. The effect sizes were small to moderate. We acknowledge that differences in means of baseline variables may reflect contextual variations, such that the assessments were done under different COVID contexts (i.e., pre COVID‐19 vs. during COVID‐19) and different stages of teacher training (start of training vs. final placement).

**TABLE 2:** Study 1: Descriptive statistics and intercorrelation of variables at time 1.

Variables	*M*	*SD*	1	2	3	4	5	6
1.Resilience	66.89	10.48						
2.Scrambled sentences test	0.72	0.23	**0.56**					
3.Recognition test	0.23	0.72	0.21	0.27				
4.Perceived stress	18.47	6.52	**−0.47**	**−0.69**	**−0.43**			
5.Reappraisal	28.86	5.16	**0.36**	**0.35**	**0.46**	**−0.35**		
6.Suppression	13.76	6.03	−0.31	**−0.35**	−0.28	0.26	0.01	
7.Stress response	7.72	4.72	**−0.32**	**−0.54**	**−0.43**	**0.67**	**−0.33**	0.16

*Note:* Significant correlations after applying the Bonferroni correction (< 0.007) are in bold.

**TABLE 3:** Study 2: Descriptive statistics and intercorrelation of variables at time 1.

Variables	*M*	*SD*	1	2	3	4	5	6
1.Resilience	70.22	11.98						
2.Scrambled sentences test	0.74	0.20	**0.46**					
3.Recognition test	0.43	0.55	0.21	**0.34**				
4.Perceived stress	18.78	7.33	**−0.50**	**−0.56**	**−0.30**			
5. Reappraisal	28.52	6.19	**0.28**	**0.28**	0.22	−0.21		
6. Suppression	6.88	5.58	0.05	−0.18	−0.17	0.16	0.12	
7. Stress response	4.50	4.72	**−0.39**	**−0.49**	**−0.25**	**0.74**	−0.20	0.08

*Note:* Significant correlations after applying the Bonferroni correction (< 0.007) are in bold.

A one‐way analysis of variance suggested that the difference in mean scores of stress response [*F* (2,176) = 0.42, *p* = 0.66], perceived stress [*F* (2,176) = 1.74, *p* = 0.02], and resilience [*F* (2,176) = 0.92, *p* = 0.40] did not significantly differ across time in Study 1. In Study 2, again there was no difference in perceived stress [*F* (2, 273) = 01.63, *p* = 0.20] or resilience [*F* (2, 273) = 2.66, *p* = 0.07] across time points. However, the difference in mean scores of stress response across time were statistically significant [*F* (2, 273) = 6.50, *p* = 0.002]. Paired *t* tests suggested that participants reported greater levels of stress at the end of their first year of teaching (T3; *M* = 9.35, *SD* = 5.02) compared to baseline, which was during the last part of training ((T1; *M* = 6.88, *SD* = 4.50), *t* (67) = 3.89, *p* < 0.001). However, the difference in levels of stress response was not significant when comparing T1 to T2, or T2 to T3.

### 3.2 Aim One: Relationships Between Stress, Positive Interpretation Bias, Emotion Regulation, and Resilience at Baseline

In Study 1, positive interpretation bias assessed via both the SST and RT were negatively associated with short‐term stress response and perceived stress, with moderate to large effect sizes. However, only the SST was positively associated with resilience. In relation to emotional regulation, reappraisal was positively correlated with resilience and negatively correlated with short‐term stress response and perceived stress, with moderate effect sizes. Suppression was negatively related with resilience, with a moderate effect size, but was not associated with short‐term stress or perceived stress.

The overall results in Study 2 replicated Study 1, but the RT and resilience measures demonstrated a weak to moderate effect, and suppression was not significantly correlated with resilience (Table 3).

### 3.3 Aim Two: Does Lower Baseline Perceived Stress, Greater Positive Interpretation Bias, and Adaptive Emotion Regulation Predict Resilience at Time 2?

Two hierarchical multiple regressions were conducted using SST or RT (Table 4). For the SST Model in Study 1, the linear combination of the covariates (i.e., age and gender) entered in step 1 did not explain a significant amount of variance in T2 resilience, *F* (2, 52) = 1.56, *p* = 0.21. In step 2, the predictor variables (interpretation bias and emotion regulation) and the covariates did not explain a significant amount of shared or unique variance in T2 resilience, *F* (6, 48) = 1.70, *p* = 0.14. The addition of the predictor variables did not improve model fit *F* (4, 48) = 11.8, *p* = 0.16.

**TABLE 4:** Study 1: Does lower baseline perceived stress, greater positive interpretation bias, and adaptive emotion regulation predict trainee teachers' resilience at time 2?

	Models											
	SST model (*n* = 55)						RT model (*n* = 53)					
	*ΔR* ^2^	*R* ^2^	*B*	*SE B*	*β*	*p*	*ΔR* ^2^	*R* ^2^	*B*	*SE B*	*β*	*p*
Step 1		0.06				0.21		0.06				0.23
Age			−0.36	0.31	−0.23	0.09			−0.37	0.21	−0.24	0.09
Gender			−1.47	2.96	−0.07	0.62			−0.56	3.05	−0.03	0.86
Step 2		0.18				0.14		0.15				0.27
	0.12					0.16	0.09					0.31
Age			−0.27	0.21	−0.18	0.20			−0.33	0.21	−0.21	0.13
Gender			−0.99	2.95	−0.05	0.74			−0.04	3.10	−0.002	0.99
Perceived stress			−0.00	0.28	−0.001	0.99			−0.23	0.26	−0.14	0.37
SST			12.09	7.68	0.28	0.12			—	—	—	—
RT			—	—	—	—			−1.12	2.67	0.07	0.68
Reappraisal			−0.07	0.28	−0.04	0.79			−0.01	0.32	−0.007	0.97
Suppression			−0.24	9.29	0.12	0.42			−0.34	0.28	−0.19	0.23

Abbreviations: RT, Recognition Test; SST, Scrambled Sentences Test.

For the RT Model, the covariates entered in step 1 did not explain a significant amount of variance in resilience, *F* (2, 50) = 1.51, *p* = 0.23. In step 2, the linear combination of the covariates and predictors did not account for a significant amount of variance in resilience, *F* (6, 46) = 1.32, *p* = 0.27. None of the predictors account for a significant amount of unique variance in resilience, and did not explain additional variance in resilience, *F* (4, 46) = 11.81, *p* = 0.31.

In Study 2, the linear combination of the covariates in the SST model (Table 5) did not explain a significant amount of variance in T2 resilience, *F* (2, 82) = 0.49, *p* = 0.61. In step 2, the linear combination of the covariates and predictors accounted for a significant amount of variance in resilience, *F* (6, 78) = 7.17, *p* < 0.001. Perceived stress and SST scores were significant individual predictors of T2 resilience, suggesting that trainee teachers who perceived lower levels of stress and had greater levels of positive interpretation bias at T1 were more likely to be more resilient at T2. The addition of the predictors in the model explained an additional 35% of the variance in resilience, *F* (4, 78) = 10.40, *p* < 0.001.

**TABLE 5:** Study 2: does lower baseline perceived stress, greater positive interpretation bias, and adaptive emotion regulation predict resilience at time 2?

	Models											
	SST model (*n* = 85)						RT model (*n* = 85)					
	*ΔR* ^2^	*R* ^2^	*B*	*SE B*	*β*	*p*	*ΔR* ^2^	*R* ^2^	*B*	*SE B*	*β*	*p*
Step 1		0.01				0.61		0.01				0.61
Age			1.60	2.87	0.06	0.58			1.60	2.87	0.06	0.58
Gender			0.13	0.14	0.10	0.39			0.13	0.14	0.10	0.39
Step 2		0.36				< 0.001		0.29				< 0.001
	0.35					< 0.001	0.28					< 0.001
Age			1.54	2.43	0.06	0.53			1.41	2.57	0.05	0.59
Gender			0.09	0.12	0.06	0.49			0.09	0.13	0.07	0.50
Perceived stress			−0.47	0.16	0.32	0.01			−0.68	0.15	−0.48	< 0.001
SST			18.21	6.22	0.34	0.00			—	—	—	—
RT			—	—	—	—			1.86	2.11	0.10	0.38
Reappraisal			−0.00	0.17	−0.002	0.99			0.09	0.17	0.05	0.61
Suppression			−0.02	0.17	0.11	0.25			0.15	0.19	0.08	0.41

Abbreviations: RT, Recognition Test; SST, Scrambled Sentences Test.

For the RT model for Study 2, step 1 results are identical to the SST Model above. In step 2, the covariates and the predictor variables also accounted for a significant proportion of variance in T2 resilience, *F* (6, 78) = 7.99, *p* < 0.001. Only perceived stress was a significant individual predictor of resilience. The addition of the set of hypothesized predictors in the model explained an additional 28% of the variance in resilience, *F* (4, 78) = 7.70, *p* < 0.001, meaning that the hypothesized variables improved the model fit over and above the covariates.

### 3.4 Aim Three: Does Lower Baseline Perceived Stress, Greater Positive Interpretation Bias, and Adaptive Emotion Regulation Predict Resilience at Time 3?

In Study 1's SST Model (Table 6), the linear combination of baseline covariates did not explain a significant amount of variance in T3 resilience, *F* (2, 45) = 0.45, *p* = 0.64 in step 1. In step 2, the linear combination of the covariates and predictors did not account for a significant amount of variance in T3 resilience, *F* (6, 41) = 2.13, *p* = 0.07. However, the SST explained a significant amount of unique variance in trainee teachers' resilience at T3, suggesting that trainee teachers who had a more positive interpretation bias at T1 were more likely to have greater resilience at T3. Although step 2 is not significant, the addition of the predictor variables explained a significant additional 22% variance in resilience, compared to the covariates alone, *F* (4, 41) = 2.92, *p* = 0.03.

**TABLE 6:** Study 1: does lower baseline perceived stress, greater positive interpretation bias, and adaptive emotion regulation predict resilience at time 3?

	Models											
	SST model (*n* = 48)						RT model (*n =* 46)					
	*ΔR* ^2^	*R* ^2^	*B*	*SE B*	*β*	*p*	*ΔR* ^2^	*R* ^2^	*B*	*SE B*	*β*	*p*
Step 1		0.02				0.64		0.04				0.45
Age			−0.58	3.04	−0.03	0.85			−2.78	2.96	−0.14	0.35
Gender			0.19	0.20	0.14	0.36			0.15	0.19	−0.12	0.43
Step 2		0.24				0.07		0.08				0.75
	0.22					0.03	0.04					0.75
Age			−0.09	2.95	−0.004	0.98			−2.35	3.21	−0.12	0.47
Gender			0.06	0.20	0.04	0.75			0.19	0.21	−0.15	0.34
Perceived stress			0.02	0.26	0.01	0.95			−0.31	0.24	−0.22	0.22
SST			20.79	7.11	0.56	0.01			—	—	—	—
RT			—	—	—	—			−0.39	2.60	−0.03	0.88
Reappraisal			−0.30	0.28	−0.02	0.29			−0.14	0.30	−0.08	0.64
Suppression			0.25	0.26	0.16	0.33			−0.01	0.26	−0.01	0.97

Abbreviations: RT, Recognition Test; SST, Scrambled Sentences Test.

For the RT Model for Study 1, the covariates did not explain a significant amount of variance in T3 resilience, *F* (2, 43) = 0.82, *p* = . 45. In step 2, the linear combination of the covariates and the predictors did not account for a significant amount of variance in resilience, *F* (6, 39) = 0.58, *p* = 0.75. None of the variables accounted for a significant amount of individual variance in resilience, and did not explain additional variance, *F* (4, 39) = 0.48, *p* = 0.75.

In Study 2 (Table 7), the covariates at step 1 did not account for a significant amount of shared variance in T3 resilience, *F* (2, 65) = 0.02, *p* = 0.98. For the SST model, the covariates and the predictor variables accounted for a significant proportion of variance in T3 resilience in step 2, *F* (6, 61) = 3.67, *p* = 0.004. The SST [*t* (61) = 2.01] and suppression [*t* (61) = 2.10] explained a significant amount of unique variance in resilience, while accounting for other variables in the model, suggesting that individuals with higher positive interpretation bias and suppression at baseline have greater levels of resilience at T3. The addition of the predictors variables explained an additional 27% of the variance in resilience, *F* (4, 61) = 5.50, *p* < 0.001.

**TABLE 7:** Study 2: does lower baseline perceived stress, greater positive interpretation bias, and adaptive emotion regulation predict resilience at time 3?

	Models											
	SST model (*n* = 68)						RT model (*n* = 68)					
	*ΔR* ^2^	*R* ^2^	*B*	*SE B*	*β*	*p*	*ΔR* ^2^	*R* ^2^	*B*	*SE B*	*β*	*p*
Step 1		0.001				0.98		0.001				0.98
Age			0.37	3.41	0.01	0.92			0.37	3.41	0.01	0.92
Gender			−0.03	0.18	−0.02	0.88			−0.03	0.18	−0.02	0.88
Step 2		0.27				0.004		0.26				0.004
	0.27					< 0.001	0.26					< 0.001
Age			0.12	3.15	0.06	0.97			−1.23	3.11	−0.04	0.69
Gender			−0.03	0.18	0.06	0.86			−0.03	0.18	−0.02	0.87
Perceived stress			−0.32	0.23	0.32	0.16			−0.69	0.19	−0.44	< 0.001
SST			16.87	8.41	0.34	0.05			—	—	—	—
RT			—	—	—	—			−5.30	2.69	−0.24	0.05
Reappraisal			0.14	0.21	−0.002	0.51			0.30	0.20	0.17	0.14
Suppression			0.50	0.24	0.11	0.04			0.38	0.24	0.19	0.13

Abbreviations: RT, Recognition Test; SST, Scrambled Sentences Test.

In step 2 of the RT model, step 1 results are identical to the SST Model. The linear combination of the covariates and predictors at baseline accounted for a significant amount of variance in resilience at T3, *F* (6, 61) = 3.65, *p* = 0.004. Perceived stress [*t* (61) = −3.60] and the RT [*t* (61) = −1.97] were significant unique predictors, suggesting that individuals with lower levels of perceived stress and *lower* levels of positive interpretation bias are more likely to be resilient at T3. The predictors explained additional variance in resilience, *F* (4, 61) = 5.46, *p* < 0.001.

In summary, in both the SST and RT model in Study 2, the linear combination of the covariates and predictors at baseline explained a significant amount of variance in trainee teachers' resilience at T2 and T3. However, this association was not found in Study 1. Nevertheless, higher SST scores (more positive interpretation bias) significantly and positively predicted resilience at T3 across both studies. In Study 2, higher RT scores (more positive interpretation bias) significantly and *negatively* predicted T3 resilience. Counter to the SST findings, greater positive interpretation bias was associated with lower resilience at follow‐up. Furthermore, suppression was found to be a significant individual predictor of resilience (in the SST Model) for Study 2; however, this was not found in Study 1.

## 4 Discussion

Two one‐year longitudinal studies investigated whether perceived stress and cognitive mechanisms measured at baseline (Study 1 assessed prior to COVID‐19; Study 2 assessed during COVID‐19) predicted trainee teachers' levels of resilience across teacher training and into their first year of teaching. In both studies, there were cross‐sectional associations between positive interpretation bias, emotion regulation, perceived stress, and resilience among trainee teachers, supporting hypothesis one. However, each variable and the linear combination of those variables did not consistently predict trainee teachers' future resilience, thus hypotheses two and three were not fully supported. Among all variables, only positive interpretation bias (measured using SST) consistently predicted trainee teachers' later resilience (with the exception of T2 in Study 1). Furthermore, in Study 2 (baseline assessed in the final placement during COVID), the linear combination of perceived stress, interpretation bias, and emotion regulation (while controlling for age and gender) consistently predicted trainee teachers' levels of resilience 8 months and 13 months later, which was not found in Study 1. The role of each predictor in resilience is discussed in further detail.

Parsons, Kruijt, and Fox (2016) proposed that individual differences in the appraisal of stressful events may play a role in one's resilience. Indeed, our results showed an association between emotion regulation (i.e., reappraisal and suppression) in trainee teachers' resilience at baseline. The results support the literature that greater reappraisal is associated with increased levels of resilience, which is adaptive in buffering against stress Troy and Mauss (2011). We found that reappraisal was negatively associated with concurrent perceived stress (Smith et al. 2019). Suppression had a negative association with resilience in Study 1, but a lack of association was found at baseline in Study 2. Nevertheless, suppression had a weak positive association with perceived stress at baseline across both studies, which is consistent with Yin, Huang, and Wang (2016).

Examining longitudinal associations, cognitive reappraisal at baseline did not have a unique role in predicting trainee teachers' resilience across time. Based on the results, we suggest that resilience interventions that solely target cognitive reappraisal may benefit from focussing on other processes as well. Unexpectedly, greater levels of suppression at baseline predicted *greater* levels of resilience among trainee teachers at the end of their NQT year (SST Model Study 2). While the relationship between suppression and resilience is inconsistent, Bonanno et al. (2004) suggest that the ability to flexibly enhance and suppress emotion in response to situational demand is associated with better future adaptation. Prior research suggests that emotion regulation is associated with one's perceived control over stressors (Troy, Shallcross, and Mauss 2013; Velardez‐Soto et al. 2022). When accounting for this finding in the context of the teaching profession, it is possible that although dealing with ongoing stressors associated with teaching may be perceived as stressful (e.g., consistently dealing with disruptive pupil behaviour), trainee teachers may not necessarily perceive those stressors as unmanageable once they have more teaching experience. More research is needed to disentangle when cognitive reappraisal and suppression can play an adaptive or maladaptive role in trainee teachers' resilience under different contexts or time points of the teaching career.

The two studies both found that perceived stress and resilience were negatively associated at baseline, which is consistent with past research suggesting psychological distress is associated with low resilience (Alatawi, Morsy, and Sharif 2022). However, levels of perceived stress did not independently predict trainee teachers' future resilience at either follow‐up timepoint in Study 1, but did for both follow‐up timepoints in Study 2. Hence, perceived stress was not a consistent predictor of trainee teachers' future resilience. We suggest that the failure to find consistent results across studies may reflect differences in recruitment contexts. Given that trainee teachers in Study 1 were not exposed to pandemic‐related stressors at baseline, potentially, mechanisms other than perceived stress, such as the presence of resources to facilitate online teaching, may have contributed more to their resilience under the pandemic. Nevertheless, the current results contribute to the literature by suggesting that efforts in reducing excessive levels of stress early in the teacher training stage, such as providing adequate support and resources to trainee teachers, or teaching stress reduction techniques, are warranted to promote future trainee teachers' resilience.

When positive interpretation bias was assessed by the SST, the overall results suggest that a positive bias was negatively associated with perceived stress and positively associated with trainee teachers' resilience cross‐sectionally across both studies. The cross‐sectional results are in keeping with Kaleta et al.'s (2023) study among general practitioners, where more positive interpretation bias (also assessed by the SST) was associated with lower levels of stress and higher levels of resilience. For longitudinal predictions, we found that the tendency to generate *more* positive interpretation bias at baseline predicted greater levels of resilience at both follow‐ups in Study 2, in keeping with Gordon et al. (2022). However, positive interpretation bias only predicted trainee teachers resilience 13 months, but not 8 months later in Study 1. The inconsistent predictability is similar to Kleim et al.'s (2014) study on medical interns whereby positive interpretation bias assessed prior to internship predicted depressive symptoms 6 months later, but not 3 months later. The authors suggest that the inconsistent predictability of positive interpretation bias could be attributable to dealing with different intern‐related stressors across time, which supports the literature that the contributing factors of resilience changes across context and time (Gu and Day 2007). It is plausible that trainee teachers may have relied on other promotive factors, such as support from school to deal with stressors associated with school placements, that were not assessed in the study.

The RT, which is often viewed as a more explicit measure of interpretation bias, failed to demonstrate a consistent pattern of findings. The RT scores were not cross‐sectionally associated with resilience in Study 1, but a small positive association was found in Study 2. Longitudinally, the RT at baseline failed to predict trainee teachers' resilience in Study 1 or at 8‐month follow‐up in Study 2. RT, however, predicted trainee teachers' resilience 13 months later (T3) in Study 2, but counter to the hypothesis, there was a negative association with higher positive interpretation bias at baseline predicting lower resilience 13 months later when other predictors are in the model. In spite of this unexpected finding, this is the first research to find that interpretation bias is a predictor of resilience among trainee teachers, in keeping with findings in relation to interpretation bias and resilience in other populations with high stress, namely women living beyond breast cancer (Gordon et al. 2022). To this point, it remains unclear whether the variation in results could be due to the use of two different aspects of positive interpretation bias measures that capture slightly different forms of positive interpretation bias. Future research should consider replicating this study in order to understand whether a more implicit and automatic measure of interpretation bias (SST) and a more conscious and explicit interpretation bias measure (RT) predict trainee teachers' resilience differently (i.e., the direction of effects) over time. Furthermore, the baseline assessments took place at different stages of teacher training across the two studies. Trainee teachers recruited in Study 2 had higher RT scores (*p* = 0.03) and higher resilience (*p* = 0.05) at baseline compared to Study 1. It is plausible that trainee teachers recruited at the final placement of teacher training (Study 2) have acquired more teaching experience, which may have enhanced their resilience to manage stressors more effectively, as opposed to trainee teachers recruited at the start of training in Study 1. Additionally, although the timing of the follow‐up assessments (8‐month & 13‐month) were spaced out the same for the two studies, we could not distinguish whether variation in results across studies were due to differences in exposure to the COVID‐19 context or a difference in the stage of teacher training.

It is possible that under certain contexts, extreme positive interpretation bias may not always be adaptive and making more negative interpretations might be beneficial in keeping with Parsons, Kruijt, and Fox (2016) model of resilience. Indeed, Notebaert et al. (2014) found that worry (or the perception of a threat) could promote or motivate adaptive health behaviours, such as engaging in sun protection behaviours. In the general occupational literature, excessively high expectations gained from work could result in disappointment (Buckley et al. 1998). In the case of trainee teachers, it is possible that making extreme positive interpretation biases in teacher training may heighten the disparity in expectations about the teaching role. For instance, researchers suggest that exposure to the reality of teaching during teaching placements may lead to early drop out of teacher training, known as ‘reality shock’ (Veenman 1984, p. 143). Chang (2009) suggested that novice teachers should learn to cope with a range of emotions and stressors they may experience prior to formally entering the teaching sector, to develop a ‘realistic view instead of an overoptimistic view of teaching’ (p. 214). Based on these suggestions, it may be that being overly positive in interpretations during the final stages of teacher training (Time 1 assessment, Study 2) may not be adaptive when working as a NQT teacher later in the NQT year (Time 2, Study 2).

In the context of trainee teachers, it may be possible that a more negative interpretation bias, as assessed by the recognition test, could motivate efforts to problem solve or engage in other behaviours to help them get support in that situation and that in turn might promote higher levels of resilience later on in their career. More research is needed to replicate and understand the current findings and determine whether this possibility is valid. In particular, whilst there were positive associations between positive interpretation bias and resilience on both types of measures (i.e., SST & RT) cross‐sectionally and at most follow‐up timepoints, it is unclear why the direction of effects between the RT and resilience was the opposite direction in Study 2 at Time 3.

Building on Parsons et al.'s (2016) cognitive model of resilience, we found preliminary evidence that trainee teachers' positive interpretation bias, emotion regulation, and perceived stress are able to predict their future levels of resilience, while controlling for the effects of age and gender. However, the mechanisms that underlie trainee teachers' resilience did not remain the same across time and context, providing support of the dynamic nature of resilience (Gu and Day 2007). For instance, perceived stress, positive interpretation bias, and emotion regulation consistently predicted trainee teachers' resilience across time in Study 2 but not Study 1. Parsons, Kruijt, and Fox (2016) suggest that a functional mapping system may play a role in guiding appropriate information processing to promote an adaptive response, such that it ‘*would take prior experiences of cognitive styles that have been effective in similar situations into account*’ (p. 300). Given that Study 2 was conducted under a continuous COVID‐19 context, where COVID‐19 was present from baseline onwards, trainee teachers may have become more capable of handling pandemic‐related stressors. The consistent pandemic context in Study 2 (vs. sudden pandemic outbreak in T2 Study 1) may explain why stress and cognitive mechanisms associate with trainee teachers' resilience cross‐sectionally and longitudinally. The introduction of novel pandemic stressors in Study 1 at T2 may have weakened the process of mapping appropriate cognitive processing styles, provided that there was limited prior experience to facilitate adaptation to a new context.

This study attempted to identify *what* processes or emotional strategies relate to trainee teachers' resilience, as well as *whether* they are strongly and consistently linked to their resilience when time and context changes. Through operationalizing resilience as both a trait and a process, the findings of this study contribute support for Parsons et al.'s (2016) cognitive model by providing further insights to understanding what factors of resilience play a more or less important role in teachers' resilience in different stages of the teaching career. This has implications for identifying appropriate resilience training targets during certain stages of teacher‐training, which might help prepare them for future work stressors. Most so‐called ‘resilience’ interventions are not developed based on resilience theoretical frameworks (Forbes and Fikretoglu 2018). Rather, most rely on stress management techniques, interventions that promote general mental health (e.g., mindfulness), or improve life skills. This might in part explain why resilience interventions tend to yield inconsistent outcomes in relation to boosting resilience.

Researchers (Forbes and Fikretoglu 2018; Leppin et al. 2014) suggested that theoretically based resilience interventions that target key resilience mechanisms that maintain and predict resilience are critical to improving the efficacy of resilience interventions. The resilience related processes or strategies identified in the present study could form targets for mechanism‐based resilience interventions. The longitudinal findings, if replicated, have potential implications for setting different intervention aims by time point and context across teacher training. For example, given that trainee teachers' level of positive interpretation bias consistently and positively predicted levels of resilience when in the newly qualified teacher year, targeting positive interpretation bias during teacher training may be a viable way to promote resilience among new teachers. This could be a promising mechanism‐based and theoretical‐based resilience intervention for trainee teachers.

Although this research has contributed to the understanding of the resilience process among trainee and newly qualified teachers, we recognize study limitations. First, due to the longitudinal nature of the study, attrition was high (T3 attrition rate was 35.2% in Study 1, 44.8% in Study 2). Consequently, participant dropout may contribute to self‐selection bias, meaning that results may not generalize to the wider trainee teacher population. Second, we could not rule out the possibility that the variation in RT scores across the two studies could reflect measurement error, due to poor attention, engagement, or understanding of the task. That said, it has been used in a number of other studies including Gordon et al. (2022). Furthermore, the contextual variation in the two studies may have played a role in the discrepancy of study results, such that the baseline assessments took place at different stages of teacher training and within different contexts of pre‐COVID‐19 and during the first year of COVID‐19. Furthermore, both studies had more female participants (72%–82%) compared to males (18%–28%). Thus, caution is needed when generalizing the results to males or other gender identities.

Through conducting two prospective and longitudinal studies, this research investigated what psychological mechanisms predicted trainee teachers' resilience over one year. This draws on and supports Parsons et al.'s (2016) cognitive model of resilience by identifying interpretation bias and emotion regulation as potential predictors of trainee teachers' resilience across time. Having found significant relationships between cognitive‐affective mechanisms and resilience in the present study, future research could explore other processes such as executive functioning. For example, Parsons, Kruijt, and Fox (2016) proposed that inhibitory control and working memory could be related to resilience. In addition, given that affective‐cognitive processes are critical to resilience (Parsons, Kruijt, and Fox 2016), future research should consider examining how stress, cognitive and affective factors jointly predict teachers' resilience across time.

To sum up, the findings of the present study support Parsons et al.'s (2016) cognitive model of resilience and set the stage for development of mechanism‐based resilience intervention for teachers. Apart from the psychological mechanisms studied in the present study, given the context and temporal dependent nature of resilience, we suggest that future research should examine whether contextual factors of resilience predict trainee teachers' resilience across time, and how these factors interact with the mechanisms identified in the present study. This would help pinpoint what factors promote trainee teachers' resilience across time. Finally, in addition to identifying modifiable cognitive processes or emotional strategies of resilience, efforts to improve contextual factors of resilience in the workplace, such as cultivating a positive school culture and providing adequate support from colleagues, are continuously and jointly needed to build teacher resilience.

## Ethics Statement

The study was approved by the university's research ethics committee (HR‐19/20‐13335; RESCM‐19/20–13335). Informed consent was obtained from all participants.

## Conflicts of Interest

The authors declare no conflicts of interest.

## Supporting information

Supporting Information S1

## Data Availability

The complete dataset generated during the current study are available from the corresponding author on reasonable request.

## Endnotes

^1^
The pandemic was underway throughout Study 2. The COVID‐19 pandemic disrupted teacher training in the UK (e.g., delaying/cancelling placements), which may have impacted trainee teachers' levels of stress or resilience. Therefore, Study 2 was done to replicate Study 1 whose baseline data was collected before the COVID‐19 outbreak.

^2^
At T2, 35 (64%) participants' placements got disrupted due to COVID‐19.

^3^
10 (21%) participants were not working as a NQT due to COVID‐19.

^4^
94 (76%) participants' placements got disrupted during COVID‐19.

^5^
10 (12%) participants were not working as a NQT due to COVID‐19.

^6^
Scoring 2.5 *SD* below the mean accuracy in the comprehension questions from the Recognition Test is an indicator of poor task performance (Hirsch et al. 2021).
