# Supplementary material for: Modifiable Psychological Mechanisms of Resilience Among UK Trainee and Newly Qualified Teachers
Source: Stress Health. 2025 Jan 21;41(1):e70005. doi: 10.1002/smi.70005 (PMC11750056; doi:10.1002/smi.70005)
Supplement: Supplementary file 1 — Supporting Information S1 [file SMI-41-e70005-s001.docx]

**Supplementary Materials**

Table S1. Data Collection Timeframes in Studies 1 and 2

| Study | T1 (*n*) | T2 (*n*) | T3 (*n*) |
| --- | --- | --- | --- |
| 1 | Sep-Dec 2019 (74)  Start of teacher training  Before COVID-19 | May-Jul 2020 (55)  During final placement year  During COVID-19 | Oct-Dec 2020 (48)  During1^st^ term of NQT year  During COVID-19 |
| 2 | May-Jul 2020 (123)  During final placement  During COVID-19 | Jan-Mar 2021 (85)  During 2^nd^ term of NQT year  During COVID-19 | Jun-Aug 2021 (68)  Near the end of NQT year  During COVID-19 |

*Note.*NQT = Newly Qualified Teacher

Table S2. Study 1: Descriptive Statistics and Intercorrelation of Variables at Time 2

| Variables | *M* | *SD* | 1 | 2 |
| --- | --- | --- | --- | --- |
| 1.Resilience  2.Perceived stress  3.Stress response | 65.95  20.53  7.40 | 10.60  6.30  3.93 | **-.27**  -.11 | **-.60** |

*Note.* Significant correlations after applying the Bonferroni correction (< .02) are in bold.

Table S3. Study 2: Descriptive Statistics and Intercorrelation of Variables at Time 2

| Variables | *M* | *SD* | 1 | 2 |
| --- | --- | --- | --- | --- |
| 1.Resilience  2.Perceived stress  3.Stress response | 68.13  18.47  7.99 | 10.69  6.31  4.25 | **-.59**  **-.37** | **.72** |

*Note.* Significant correlations after applying the Bonferroni correction (< .02) are in bold.

Table S4. Study 1: Descriptive Statistics and Intercorrelation of Variables at Time 3

| Variables | *M* | *SD* | 1 | 2 |
| --- | --- | --- | --- | --- |
| 1.Resilience  2.Perceived stress  3.Stress response | 68.63  19.56  8.15 | 9.07  6.02  3.91 | **-.56**  -.26 | **.64** |

*Note.* Significant correlations after applying the Bonferroni correction (< .02) are in bold.

Table S5. Study 2: Descriptive Statistics and Intercorrelation of Variables at Time 3

| Variables | *M* | *SD* | 1 | 2 |
| --- | --- | --- | --- | --- |
| 1.Resilience  2.Perceived stress  3.Stress response | 66.26  20.37  9.35 | 11.91  6.84  5.02 | **-.58**  **-.43** | **.78** |

*Note.* Significant correlations after applying the Bonferroni correction (< .02) are in bold.
